# The Middle Ages Contributions to Cardiovascular Medicine

**DOI:** 10.5935/1678-9741.20160031

**Published:** 2016

**Authors:** André Silva Ranhel, Evandro Tinoco Mesquita

**Affiliations:** 1 Universidade Estadual Paulista (UNESP), Franca, SP, Brazil; 2 Universidade Federal Fluminense (UFF), Niterói, RJ, Brazil

**Keywords:** History of Medicine, Medicine, Arabic, Cardiology

## Abstract

The historical period called the Middle Ages, a long interval between the 5^th^ and the 15^th^ centuries, is still commonly known as the Dark Ages, especially in the area of health sciences. In the last decades, this "classic" view of the Middle Ages has been gradually modified with advances in historiographical studies and the history of science. During that period in Western Europe, knowledge about the human body suffered a regression in terms of anatomy and physiology, with the predominance of religious conceptions mainly about diseases and their treatments. Knowledge on the cardiovascular system and heart diseases has been classically described as a repetition of the concepts developed by Galen from the dissection of animals and his keen sense of observation. However, the Middle East, especially Persia, was the birth place of a lot of intellectuals who preserved the ancient knowledge of the Greeks while building new knowledge and practices, especially from the 8^th^ to the 13^th^ century. The invasion of the Arabs in North of Africa and the Iberian Peninsula and the eclosion of the Crusades resulted in a greater contact between the East and the West, which in turn brought on the arrival of the Arab medical knowledge, among others, to 12^th^ century Europe. Such fact contributed to an extremely important change in the scientific medical knowledge in the West, leading to the incorporation of different concepts and practices in the field of cardiovascular Medicine. The new way of teaching and practicing Medicine of the great Arab doctors, together with the teaching hospitals and foundations in the Koran, transformed the Medicine practiced in Europe definitely. The objective of this paper is to describe the knowledge drawn up from the Middle Ages about the cardiovascular system, its understanding and therapeutic approach to cardiologists and cardiovascular surgeons.

## INTRODUCTION

The Middle Ages is the time period commonly defined between the 5^th^ and 15^th^ centuries, from the fall of the Western Roman Empire to the fall of Constantinople. It relates mainly to Western Europe, but it is also extended to the Byzantine Empire and the Arab kingdoms in the East. The medieval period was given this name by the intellectual people of the 16^th^ century. Eager to revive the Ancient History and its knowledge, they defined the Middle Ages (from the Latin media *aetas, medium aevum*) as the period of time between theirs and the ancient ones. Quickly, it was called the Dark Ages because of religious predominance, especially by the Christians in the West, as well as the alleged delays in several areas of knowledge and the precarious living conditions of the time^[1,2]^.

This period fits into the models of ancient society, related to quality and expectancy of life. It is typical of pre-industrial societies, since there were high birth and death rates, which suffered variations from insurgencies or facts contrary to population growth, such as: plagues, climate change, wars and food shortages. Three great epidemics weakened Medieval Europe. The epidemics of malaria between the 3^rd^ and 5^th[1]^ centuries; the plague between the 6^th^ and 8^th^ centuries^[3]^, reducing the population level in Europe to the lowest number since the second century; and the Black Death, between the 14^th^ and 17^th^ centuries, which caused the death of 1/8 to 2/3 of the European population, depending on the region^[1]^. Although it is difficult to have reliable statistical estimates in the period due to the precarious sources, life expectancy was between 20 and 30 years and the mortality rate was about 30 and 40 thousand individuals a year, mainly because of epidemics^[4]^.

Medicine in the Western Middle Ages gained new forms with the religious predominance of Christianity. Body and soul are interconnected in a complex philosophical and theological dualism. Diseases and body ailments are seen as consequences of sins inflicted to God, as the classic example of leprosy, present in the Scriptures^[2,5]^. Thus, the act of taking care of patients becomes an obligation of religious people (nuns, monks and clergy), the medical practice becomes an act of charity^[6]^ and hospitals become places which provide more comfort than cure^[5]^. However, sick people were also treated at a distance and in fear because of the possibility of contagion, both physical and spiritual. In this context, the barber-surgeons, healers and women who manipulated herbs were increasingly required for medical treatment, even by religious people. Medical practice in the West only becomes academic when doctors are graduated from universities, from the 12^th^ century on. Even with universities and physicians with academic knowledge, only after long efforts, between the 13^th^ and 15^th^ centuries, the medical profession became more valued and more required than the healers and barber-surgeons^[7]^.

Medicine theoretical knowledge was based on Galen's formulations (four humors) and extrapolations about anatomy and physiology of the cardiovascular system from animal dissections^[8]^. Galen's ideas were incorporated and considered dogmas by the Catholic Church. Faith and religious rituals were the basis to face diseases and epidemics, besides promoting rituals and attitudes which involved prejudice, "witch hunts" and hysterical behavior by the heavily present society in the Middle Ages (10^th^-15^th^ centuries). Gradually, the Western world renewed its knowledge in different fields, particularly in Medicine, as a result of the scientific and cultural splendor happening in the Islamic world. Such scientific splendor comes to Europe through the knowledge generated by the Arabs, in the reading of Greek texts and new ways to conduct medical training and patient care, while in contact with the Arab world. The purpose of this review is to present the knowledge created in the Middle Ages on the cardiovascular system and also the advances in understanding diseases and approaches that emerged in the Middle Ages.

## THE WESTERN MIDDLE AGES AND GALEN'S VIEW

### Middle Ages and Medicine

The Middle Ages is commonly divided into two periods: the Early and Late Middle Ages. The first period, characterized as the time interval from the 5^th^ to the 10^th^ century, is marked mainly by the formation of Germanic kingdoms in Europe, the expansion of Christianity, the founding of the Catholic Church as an institution, along with the organization of the Carolingian Empire (800-888) and the formation of the Christian kingdoms, arising from their fragmentation. With the emergence of the Carolingian Empire, suzerain-vassal relations are developed, as well as feudal society. The population level in Europe suffered a decline because of food shortages and the spread of the plague, being gradually restored at the end of this period due to further exploration of fields and forests for agriculture. The High Middle Ages is marked by political stability of the Byzantine Empire in the East, the rise of Islam, and the Arab expansion into Christian territories^[1,9]^.

On the other hand, the Late Middle Ages, the period between the 11^th^ and 15^th^ centuries, is marked by several social, economic and political changes in Europe. There was a strengthening of the feudal society between the 11^th^ and 13^th^ centuries, predominantly agrarian and stratified ones, politically defragmented and under the Church's cultural and social control. However, at the same time, there was increased population growth, development and spread of cities all over Europe, great cultural development in arts, literature, education, philosophy and science. This time is also marked by a closer contact between East and West, through the Crusades, trade relations, as well as religious and secular expeditions in order to know and absorb the knowledge of the Arab people. The final centuries of the Middle Ages (14^th^ and 15^th^) are marked by political and religious crises, the plague outbreak and the European territorial expansion (commercially with Italians and territorially with the reconquered wars against Muslims), which promote and permeate great changes that mark the following centuries^[1,9]^.

The medical practice itself can be verified in the temporal extents of the Middle Ages. Before the 13^th^ century, the practice of curing the body was closely connected to that of curing the soul, considering the most identified diseases as physical expressions of sins. This way, the task of taking care of the sick people is relegated to the religious, including monks, nuns and secular clergy, and to the laypeople who had practical experience on how to deal with the sick. Hospitals are institutions that took a long time to be built in medieval Europe, except for Spain, under Muslim control. It can be noticed, before the 13^th^ century, the presence of specific places in monasteries to deal with itinerant sick people and monks, and also the houses of God (*domus Dei* in Latin, *hôtel - Dieu, maison -Dieu, hôpital or hospice* in French), places where the major concern was the person's soul salvation, not the cure of the body itself. Even after the existence of hospitals, from the 13^th^ century on, such places were considered a place to provide more comfort than the cure itself^[5-7]^. Doctors, as professionals, also arise after the 12^th^ and 13^th^ centuries, with the creation of universities and the breaking of knowledge monopoly practiced by the monks. Until then, the School of Salerno, founded in the 10^th^ century, was one of the few learning centers in Europe for medical practices. Despite graduating from universities, doctors had to make a great effort for their academic and practical knowledge to be recognized as the only ones viable in patients' treatment, constantly fighting against the practice of healers, barber-surgeons, superstitious and quacks, until the end of the Middle Ages^[7]^.

### Galen's Knowledge

**Fig.1:**
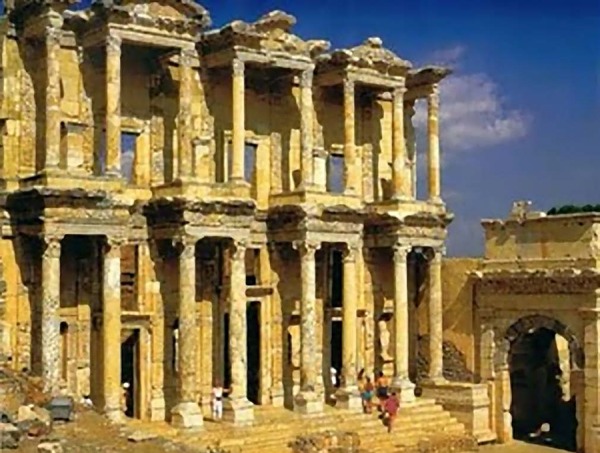
Ruins of the ancient Library of Alexandria.^[12]^

In the field of Medicine, specifically the one related to the knowledge of the cardiovascular system, the Middle Ages could see the hegemony of the knowledge created in the 2^nd^ century by Claudius Galen (129 - 199?). Originally from Pergamum, a Greek city under Roman rule, Galen made use of the majestic city library, with more than 200 thousand books, to study all the philosophical, scientific and Greek Medicine literature of the time. Galen also made use of the information stored in the School of Alexandria, the main learning center of the time, where he could find the largest library of the Ancient times and complete skeletons, thereby allowing him to study the human body thoroughly. Then, he became famous in Greek Roman Medicine^[8,10,11]^ (Figure 1).

**Fig.2:**
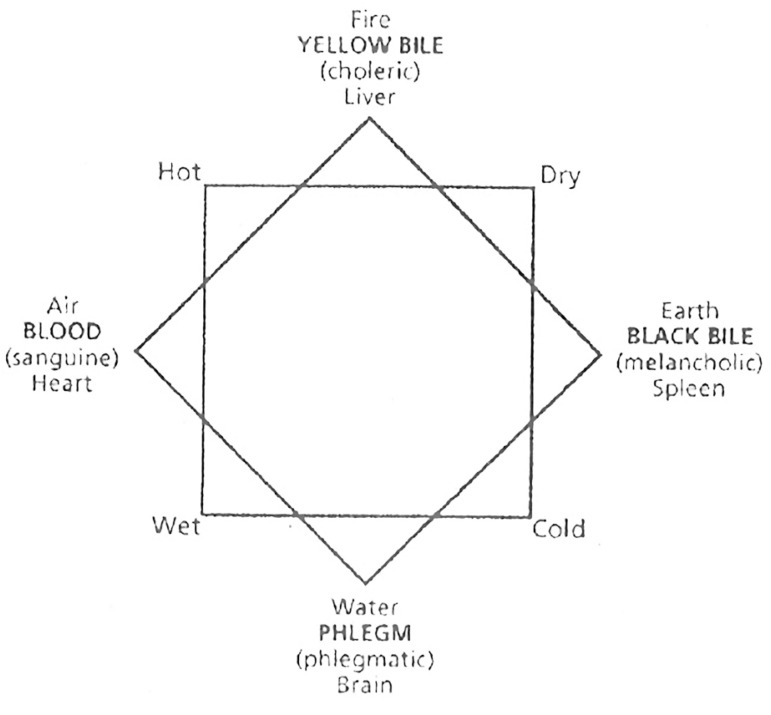
Scheme of Galen's humoral theory.^[10]^

Galen treated several kinds of people, from gladiators to emperors. He revolutionized the field of Medicine in his time through observations on the human body, systematization of previous medical knowledge based on such observations, experimentation and studies involving dissections of animals, like pigs and monkeys. He was an heir of Hippocrates' Humoral Theory, reaffirming the existence of four fluids, or humors, in the human body: blood, black bile, yellow bile and phlegmatic. Such humors were divided into proportions for the body and the necessary harmony among them resulted in perfect health. But, Galen associated the humoral theory to Aristotle's ideas, which stated that the basis of existence resided in four elements: water, air, earth and fire. This way, each vital organ would have the predominance of one type of humor, which in turn, would be related to a natural element. Each humor would then have its temperature related to the nature element that it corresponded to, deriving the four temperaments: blood, phlegmatic, melancholic, choleric. For Galen, all diseases of the human body were the result of a temper, which excelled the others, caused by the disharmony of humors^[8,10,11]^ (Figure 2).

Galen's theories were ahead of his time, permeating throughout the medieval period and still consulted until the middle of the 18^th^ century. Moreover, during the Middle Ages, Galen was recognized as an indubitable name concerning the knowledge of the human body, being widely accepted by the Catholic Church and studied by religious scholars, despite being Greek-Roman and non-Christian. This is due to his heritage of Aristotelian thinking, which stated that everything had a first cause, that could be identified and solved, and everything would have an end because of this cause. His affirmation comes from this idea, valid until today, that every change in body function is the result of an injury and every injury involves a change in body function. Christianity soon adapted this idea, identifying God as the cause of all things and final salvation as the ultimate end of everything. Therefore, all things and events were in God's perfect plan, and nothing happened by chance. Another fact that facilitated such adaptation of Galen's thought, his acceptance and diffusion of Christianity, was his belief in a single deity with the power to interfere spiritually in the material world. Thus, the human body would be simply an instrument of the soul, guided by this single deity. Due to such conceptions, Galen's teachings could be accepted by the Catholic Church as well as by Arabs and Jews, making the church reject any healing practice not endorsed by Galen Medicine^[6,8,10,13,14]^.

**Fig.3:**
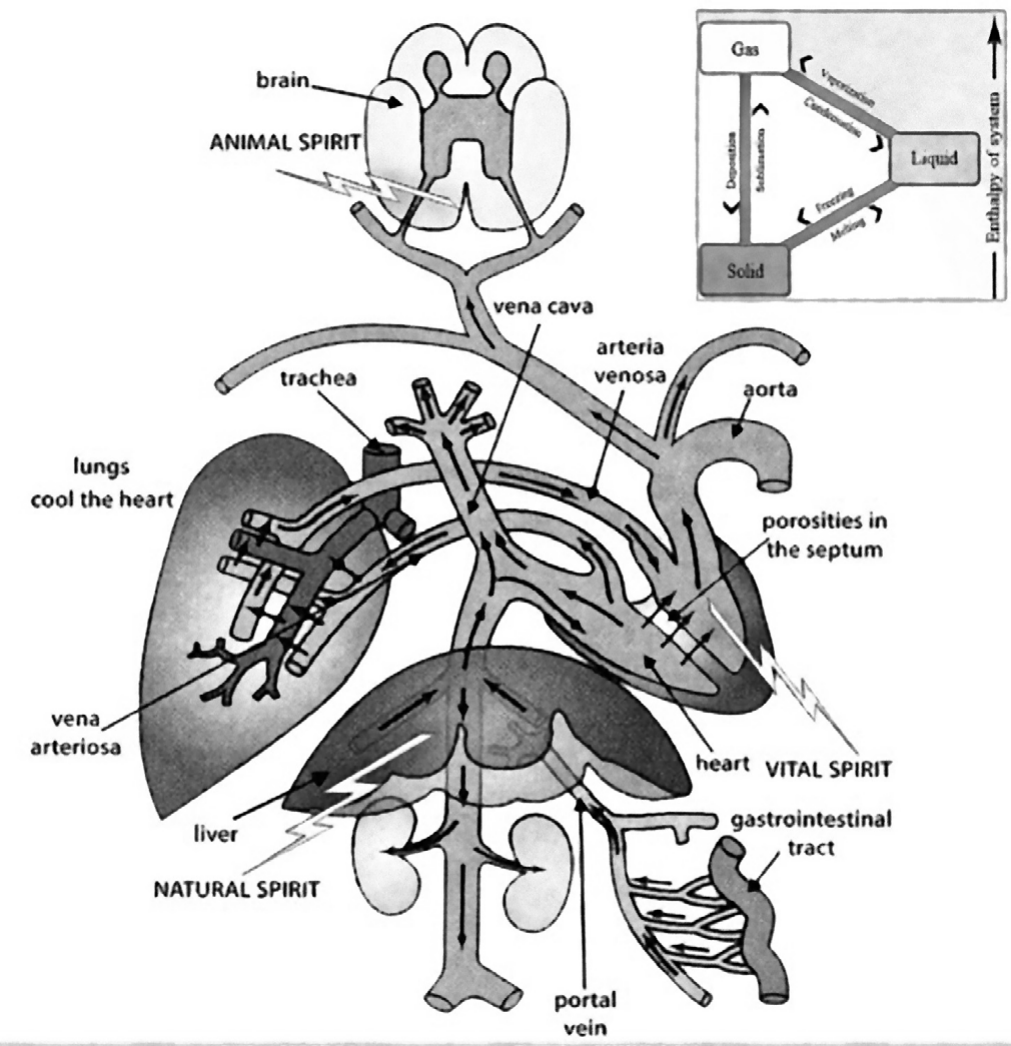
Galen's Tripartite Scheme, explaining how the blood circulated through the body.^[14]^

Some of Galen's advances were remarkable, such as the identification of the presence of blood in the arteries rather than air, the discovery of blood circulation in veins and arteries, and the existence of two ventricles in the heart, each one responsible for a different function in the circulatory system. Based on blood circulation, Galen identified the distribution of humors around the body in addition to the fact that the heart has the function of feeding the whole body with *pneuma* or spirit. Blood, full of impurities, would go from the right side of the heart to the lungs to be purified by breathing and then return to the right side. As a result, he identified two major veins in the right ventricle responsible for this transport (pulmonary artery and vena cava). After purification, the blood would pass from the right side to the left by small invisible pores, there it would be again combined with air and *pneuma*, then it would be distributed throughout the body; here, he identified the aorta and the pulmonary veins on the left side. In addition, Galen gave great importance to the liver, which would turn digested food into blood and then send it to the heart to be filtered. Despite achieving great advancements regarding blood circulation throughout the body, Galen said that the venous and arterial systems were completely separated and not connected^[11,13,14]^ (Figure 3).

In short, Galen promoted great advances concerning knowledge on the anatomy and physiology of the heart. However, he made some mistakes, most of them because of ancient knowledge reproduction and the few opportunities for human body dissection. His progress was essential and permeated a great part of the knowledge present in medieval Western Europe until the 16^th^ century, but his mistakes were also reproduced, such as the presence of pores connecting the right and left ventricles, the separation between the blood and venous system, heart and blood function to distribute the spirit throughout the body and the humoral theory, stating that all diseases and evils would come from the disharmony between the amount of humors in each part of the body^[8,13]^.

## ARAB MEDIEVAL MEDICINE: THE INFLUENCE OF QU'RAN AND THE GREAT MASTERS

A great part of the mistaken view still present about medieval history being considered a period without scientific advances results from little knowledge on the contributions of the Late Middle Ages, in particular the contributions of Arab medieval Medicine.

### Panorama of the Arabs in the Middle Ages

**Fig.4:**
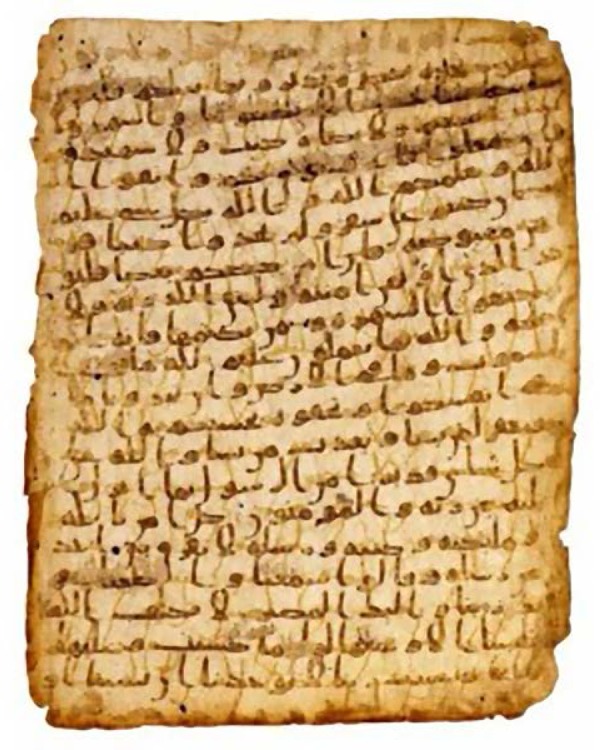
Qur'an page of medieval times.^[15]^

Islam has its origins in the 17^th^ century Arabian Peninsula. Until then, the Arabs were divided into several tribes, most of them were polytheistic religious nomads, whose main common cultural trait was their language and oral traditions. Muhammad (570-632), who belonged to one of those tribes, was a caravan merchant for most of his life. From the age of 30 on, he started to receive revelations from Angel Gabriel. Those revelations were orally transmitted among the Arabs and later formed the holy book of Islam, the *Qur'an* (Figure 4). Strongly influenced by Persian Zoroastrianism, Judaism and Christianity, Islam was established as a new monotheistic religion, eschatological, based on the constant struggle between good and evil^[9]^. Muhammad managed to unify the Arabs, a fact that contributed to the subsequent creation of the Islamic Empire, divided into Caliphs, and unification of the Arabian Peninsula. The Islamic territorial expansion was fast and intense. In the 7^th^ century, the whole Arabian Peninsula, Middle East, West India and North Africa were already dominated by Islamic Caliphs, followed by the Iberian Peninsula conquest in the 8^th^ century^[9]^. Christians were able to recover territories in the Middle East which were under Arab rule, especially after Jerusalem's conquest in the First Crusade in 1099. However, Christian ruling in the Middle East lasted only until 1187, with the conquest of Jerusalem by the Arab Sultan Saladin^[9,16]^. The Arabs lost their control of the Iberian Peninsula after the long reconquest wars initiated by the Christians, which lasted from the 8^th^ century to 1492, when they finally regained the territories of what is now Portugal and Spain. However, the Muslim expansion continued on, with the muslim Turks being subjugated to the Byzantine Empire, the Christian empire in the East, and earning the capital Constantinople in 1453, thereby marking the end, for many historians, of the Middle Ages^[9]^.

### The Philosophical and Religious Influence in Arab Medicine in the Middle Ages

The East had always witnessed a certain advancement in Medicine compared to the West. The first school-hospitals were founded in Persia by the Nestorians, firstly founded as a Gondishapur school with a large hospital connected to it, a practice which spread all over the Middle East^[8]^.

The Arab medical schools could preserve ancient knowledge about medical arts, mainly the Greek one, keeping, translating and copying essential pieces of work, such as Hippocrates, Aristotle and Galen. In those schools, where we could find both Arabs and Jews as well as Christians and Muslims of various ethnicities, fragmented and dispersed knowledge of ancient authors was systematically organized and translated, producing a wide understandable and accessible medical literature in Arabic. This classic literature was constantly updated with new documents written by the Arab doctors, based on both readings and experiences of medical practice in school-hospitals. This way, as the basis of medical conceptions remained according to Hippocrates humoral theory perpetuated by Galen, and the knowledge of human anatomy remained limited due to the prohibition of human body dissection, many innovations about the knowledge of the body and disease treatments were carried out due to observation and experimentation^[8,17]^.

It may be pointed as a cause of such advance, the medieval Arab contact with the Hellenistic culture, especially regarding philosophy. When Islam replaced Christianity in the East after the 8^th^ century, the tradition of reading the Greek classics could be observed in medical schools, where people were taught mathematics, philosophy, theology and jurisprudence in addition to Medicine. The result was an increasing contact with Aristotelian philosophy and the consequent enhancement of the sensible world, considered a possible world to know, contrary to what happened in the Christian West in the same period of time, where the sensible world was wrong and imperfect. Thus, for the Arabs, the body knowledge, in its anatomical and physiological form, is considered extremely important, whereas according to Christianity, until the 12^th^ century, the body is just the place for sins and its sickness would only come from the spiritual world. However, Arab philosophy mixed with theology, and ultimate knowledge would come from God^[18]^.

On the other hand, even regarding the religious aspect, we notice some facts that contributed greatly to the development of Arab Medicine. The two sacred Islamic books which compose the Sacred Law, or *Shariah* (path), are the *Qur'an* (revelation given directly by Allah to Muhammad) and the *Hadeeth* (practical lessons pronounced by Muhammad). Here, we may see a great importance given to medical knowledge. Arab teachers and doctors encouraged and constantly searched for new knowledge and research that resulted in major discoveries based on the sacred writings. Every disease had been created by God and He had created a cure for each of them, not only cures and spiritual treatments, but also the treatment received by doctors was encouraged, since it was one of the ways whereby God provided the cure for human diseases. Most of the teachings regarding healing and disease prevention contained in the sacred books refer to lifestyle, food and personal hygiene habits, besides containing some guidelines on minor surgeries. For all that, the appreciation of doctors as healers authorized by God allowed for the construction of new knowledge about the anatomy and physiology of the human body, as well as diseases and their treatments discovered by Arab doctors^[19,20]^.

In addition to information on treatments and cures of diseases in general, the sacred writings bring great information about specific heart diseases, and also physiological and anatomical information. The heart is considered the center of emotions, actions, intentions, desires and knowledge, and, many times, the diseases are related to the emotional state (anger, fear, aggression, etc.) and even to the spiritual one (life in sin, blasphemy, unbelief).

Nevertheless, besides the spiritual view, there are several references of an anatomical knowledge of the heart, like a muscle, as in the passage in which Angel Gabriel would have performed a surgery on Muhammad, extracting a blood clot from his heart. There is also reference to the knowledge of veins and arteries and their vital importance, because the sacred books say that God created man's life and could take it, cutting either his jugular vein or the *Al- Watin* (aorta)^[19]^.

Such references about medical concepts in the sacred books can be derived from the contact Muhammad had with doctors graduated from school-hospitals. Many of the Arab tribe nomads attended those hospitals, like the Gundishapur one, in order to go back to their tribes and treat the sick people there. Muhammad had great contact with the doctor al-Harith bin Kalada, who influenced him mainly on hygiene notions present in the *Qur'an*^[9]^.

### Great Arab Doctors and their Contributions to Cardiovascular Medicine

**Fig.5:**
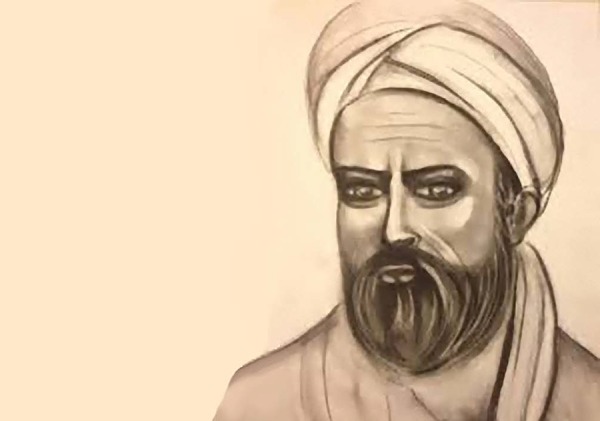
Haly Abbas.^[21]^

Medieval Arab Medicine culture, especially the one developed between the 8^th^ and 13^th^ centuries, provided the advancement of medical science knowledge at school-hospitals; this knowledge was based on ancient writings and practical experiences, supported by philosophical and religious bases^[9]^. Besides the general field of Medicine, there was a great development in knowledge about the heart, regarding its anatomy and physiology, diseases and their treatments. To demonstrate the scientific advancement of that time, we have chosen three Arab doctors who showed important contributions to the knowledge of the cardiovascular system: Haly Abbas (? 930-994), Avicenna, or Ibn Sina (980-1037) and Ibn al-Nafis (1210-1288) (Figure 5).

Haly Abbas was a Persian physician of the 10^th^ century, who lived at a time between two great names of Arab Medicine: Rhazes and Avicenna. He was the court physician of Adud al-Dawla-Fana Khusraw (936-983), king of Persia, and also at Azodi Hospital in Baghdad, where he wrote his most important piece of work "The Complete Book of Medical Art" (*Kamil al-al sina'ah-Tibbīyah*), or "The Royal Book" (*al-Kitab al-Maliki*). It is in this encyclopedic book that we can find the greatest references by Haly Abbas concerning the knowledge of the heart, in which we notice his remarkable effort to reject some of Aristotle's and Galen's outdated ideas. On the question of the venous and arterial system, Haly Abbas made the first distinctions between veins and arteries based on their thickness, in addition to a detailed description about the descending aorta of the thoracic structure^[13,21]^. Yet, his greatest contribution was making one of the first mentions about a connection between the venous and arterial system, describing it in the following way in the "Royal Book": "*...There are some foramina within the non-pulsating vessels [veins] that open to the pulsating vessels [arteries]...*"^[21]^.

Furthermore, he made great advancements in the anatomical description of the heart, although not with the nomenclature being used nowadays. He described the heart as two main chambers, one on the right and another on the left, the latter being where the arteries would have their origin, and considering the liver the origin of the veins. He also recognized the existence of two atria and two auricles as well as the aortic and mitral valves, describing their characteristics and the presence of the pericardium^[13,21]^.

**Fig.6:**
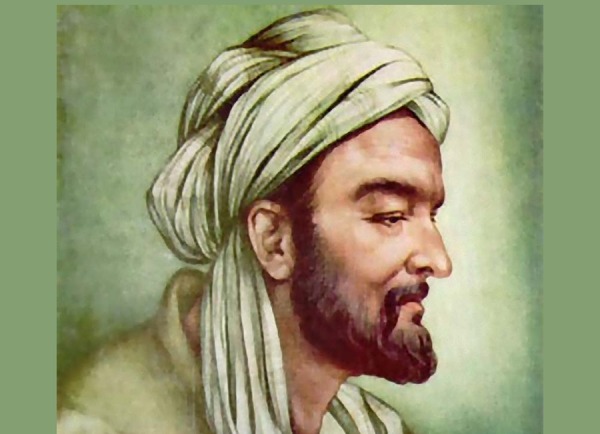
Avicena.^[21]^

The great Arab master Avicenna promoted what we consider the biggest set of developments related to medical science, in particular on the knowledge of the cardiovascular system, and he was the most influential Arab intellectual person in both the East and the West. He was also originally from Persia. Avicenna was exceptional, having memorized the entire Persian literature at 10 years old, including the *Qur'an*, and becoming a famous doctor at the age of 18. He wrote more than 450 books, namely about astronomy, logic, philosophy and Medicine treaties. Avicenna wrote all this literature living a troubled life of persecution and escapes due to his political opposition to the Persian government. His major works on Medicine are: "Canon of Medicine" (*al- Qanun fi al- Tibb*), "The book on drugs for cardiac diseases" (*Kitab al- Adviyt al- Qalbiye*), and the less well-known "Book on Pulsology" (*Resaley and -Ragshenasi*). He made great progress in the anatomical knowledge of the heart, although he had accepted some of Galen's misconceptions, such as the existence of pores connecting the two ventricles of the heart, allowing the passage of blood from the right to the left side. He recognized the origin of the arteries on the left side of the heart and veins in the liver; moreover, he identified the difference between the thickness of the left and right ventricle walls. In addition, he was the first to mention the difference in atria and ventricles contractions, as well as the existence of capillary circulation^[22-26]^ (Figure 6).

**Fig.7:**
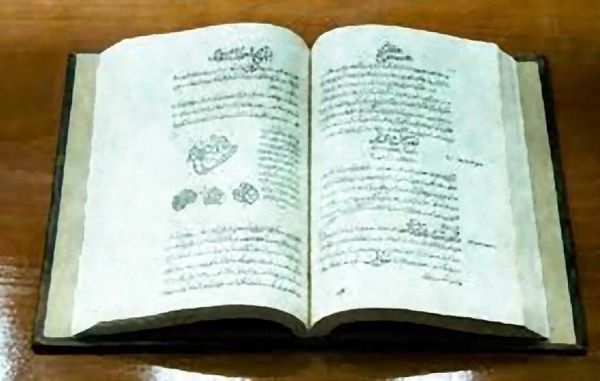
Persian calligraphy copy of the Canon of Medicine.^[28]^

Avicenna was greatly influenced by Hippocrates' and Galen's humoral theories, and reproduced much of such theories in his writings, especially when he made descriptions of several diseases. However, even using humoral theory as a basis, it is evident the advancement of theorizing and observation of heart ailments made by Avicenna. Atherosclerosis was identified by him, though not by this name, because it was recognized that the abnormal accumulation of moods in the veins and other areas could cause obstruction, and the worst obstructions are those which happen in the arteries of vital organs, such as the brain, heart and liver. Vasovagal syncope was also observed by Avicenna, although it received the name of *al- Lawa*. He discovered that patients who had tiredness, fatigue and flushing suffered from a humor disharmony during the distribution of humors throughout the body through the blood. According to Avicenna, there would be a predominance of bile, warm humors in the heart, and the brain would be predominantly cold with the phlegm, cold humor. The *al-Lawa* would be the result of poor humor distribution around the body, with an excess of black and yellow bile, hot humors that caused brain malfunction, being sent to the brain. This would result in tiredness, fatigue and flushing, nowadays recognized as vasovagal syncope symptoms. Nevertheless, not all diseases referred to humor disharmony, such as palpitation, identified by Avicenna as a heart physiological distress caused by lesions in its external coating or in organs next to it. Palpitation, when it became acute, caused fainting, and when it became constant could cause death as well^[23-25]^ (Figure 7). 

Avicenna was also a pioneer in relating a patient's pulse rate with evils and internal feelings, thereby advancing in the studies of the arterial pulse and being the first to measure the wrist pulse. He identified several circulation changes related to the patient's conditions, such as age, gender, drinking and food consumption, anger, fear, pregnancy, diseases and even in relation to weather conditions. Many recent studies have proven those associations^[29-32]^. In the end, Avicenna also ventured into the field of Medicines to cure diseases. His book about drugs for heart ailments has countless composition forms, some of them have effect on the cardiovascular system and are currently established as the 'zarnab' drug, which is a calcium channel blocker^[29]^.

**Fig.8:**
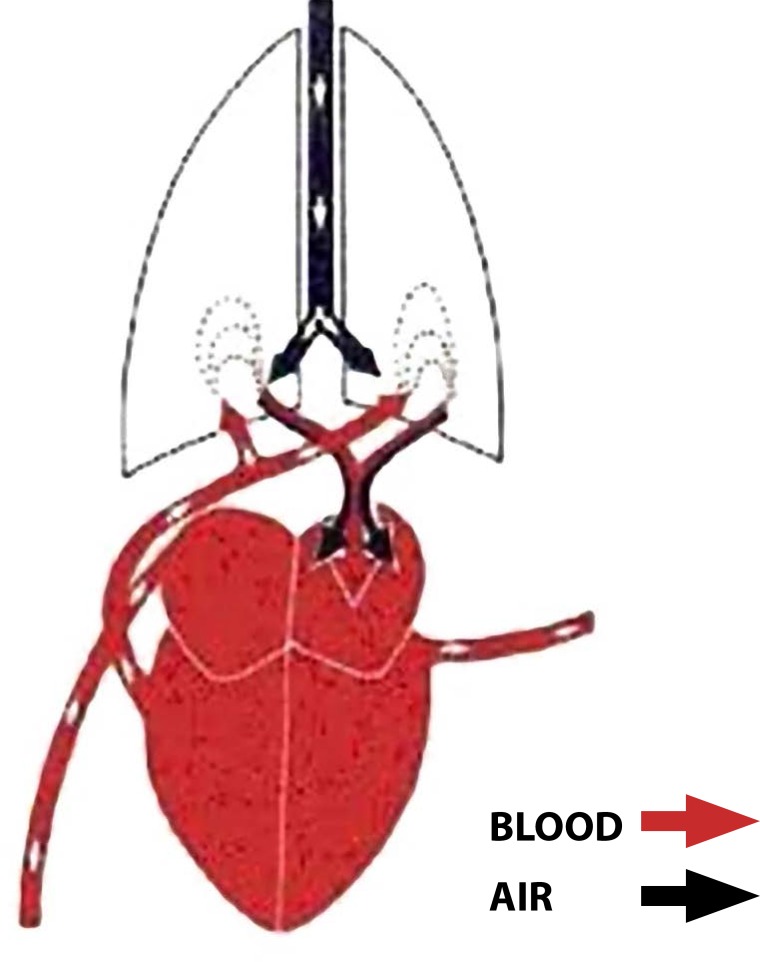
Illustration of the minor circulation of blood according to Ibn al-Nafis.^[35]^

Finally, we present one of the less well-known Arab doctors of the 13^th^ century, who made perhaps the most important and surprising discovery about the heart anatomy and physiology. Ibn Al-Nafis was born in 1213 in Damascus. He studied at the school-hospital in the city. Physician and professor in Egypt, he was the author of several pieces of work, the most important ones being "The Comprehensive Book on the Art of Medicine" (*Kitab al-Shamilfi 'l-Sina'a al-Tibbiyya*), which consisted of 300 volumes, though it was not completed, and the "Commentary on Anatomy in Avicenna's Canon" (*SharhTashrih Qanun al*), comprised of 80 volumes. Ibn Al-Nafis stands out for the clear criticism of Galen and Avicenna, pointing out the flaws and mistakes in their theories, which was something almost unthinkable to be done at a time when the ancient writings represented the truth. Most likely he had dissected human bodies to have his greatest achievement: the discovery of the pulmonary circulation^[33-35]^ (Figure 8). 

Ib-Al-Nafis described the pulmonary circulation like this: "*The blood from the right chamber of the heart must arrive to the left chamber, however, there is no direct pathway between the two sides. The thick septum of the heart is not perforated and has no visible pores as some people thought (referring to Avicenna) or invisible pores (in reference to Galen). The blood from the right chamber must flow through the vena arteriosa (pulmonary artery) to the lungs, spread through its substance, be mingled with air while in the lungs, pass through the arteria venosa (pulmonary vein) to reach the left chamber of the heart to form the vital spirit*"^[33]^.

Moreover, he was the first to describe the role of coronary arteries, which are truly responsible for nourishing the heart^[33]^.

### The Late Middle Ages: the Arrival of Arab Medical Scientific Knowledge and its Transformations

All this knowledge produced by the Arab masters, added to the various translations of the Greek classics into their language, reached the Christian West and changed deeply the way of teaching and practicing Medicine. The most remarkable contact between the Christian West and the Muslim East was clearly warlike. The crusades greatly marked the way of thinking of the time, causing great aversion to Islam in Europe, especially among the religious ones. However, the contact with Arab culture as a result of the Crusades conduced to a search for Arab works and their translations in order to better understand the enemy against whom the Christian West fought^[17,36]^.

Italy had already flourished in business since the 11^th^ century, keeping constant trade with the East, and the Muslim-ruled Iberia was quite open to contact with the European Christians, considering they were coming from a tradition of tolerance toward Christians and Jews within their own boundaries. It was through those commercial and cultural contact centers that Arab scrolls entered the Western Christian world, being translated into Latin and influencing many intellectuals of the time^[36]^. Particularly noteworthy is a doctor called Constantine, the African, who worked for forty years in Syria, India, Egypt and Ethiopia before taking part in the doctors' and teachers' group at Salerno school, the first medical school in Europe. He came up with several manuscripts, extensive background and medical techniques^[9]^. Thus, Greek classics which were forgotten for a long time in the West, such as Hippocrates', Galen's and Aristotle's, as well as new writings made by the Arabs in the fields of philosophy, arithmetic, Medicine, among others, entered the medieval Christian area early in the Late Middle Ages. During this period of time, between the 12^th^ and 13^th^ centuries, Europe experienced an outbreak and growth of cities, the creation of the first universities and a greater knowledge secularization. Arab works, then, were read by intellectuals, scholars and even religious people, modifying gradually the philosophical and scientific conceptions of medieval Europe^[9,36]^.

The reading of Arab medical works and the change in the intellectual scene in Europe generate important transformations in Medicine at the time. Gradually, professionals who had only practical knowledge go to universities in search of greater knowledge. During the 13^th^ and 15^th^ centuries, the graduated doctors fought against healers and barber-surgeons, with the purpose of being recognized as the most prepared ones to deal with patients. Moreover, it is when the need for body anatomy knowledge in medical training arose in Europe. In 1240, Emperor Frederick II, of Naples, insisted that it was necessary for the surgeons to have anatomy training. However, the first milestone in the history of public dissections at universities, in terms of teaching, occurred only in 1315, in Bologna, by physician Mondino De' Luzzi; the same happened in Montpellier, only in 1376, and in Paris in 1407. Despite being discreet and facing various religious difficulties, this first step towards an observation science marked the beginning of progress made in the Renaissance in Italy, mainly in the medical field^[8]^. Therefore, the great names of the Renaissance, such as Leonardo Da Vinci, Michael Servetus, Andreas Vesalius and William Harvey could dissect and observe corpses, making great advances in Medicine and especially on the knowledge of the cardiovascular system^[9,21,37]^, probably being great readers of Arab works on the subject. Michael Servetus in his "*Christianismi Restitutio*" work of 1553 talks about Ibn Al- Nafis' great advances regarding pulmonary circulation, compiling part of his work. But, not mentioning the Arab author, pretending to be the original author of the groundbreaking discoveries until not long after finding Ibn Al- Nafis' books^[9,33-35]^.

## CONCLUSION

Medicine and cardiovascular science in the medieval times, rather than presenting a great stagnation, received important contributions from philosophical ideas, as well as Arab medical knowledge. Arab medical science had already made great contributions in the sacred books of Islam, in particular on disease prevention and the cardiovascular system anatomy. At the same time, bright doctors and teachers, especially Avicenna, revolutionized cardiovascular knowledge, founded and taught at the first school-hospitals, and refuted the traditional knowledge present in Galen's work. During this time, when Europe remained under the aegis of the Catholic Church, it received strong and definite influence from the Arabs, such as the creation of the first medical schools and universities. That influence provided advances concerning the knowledge of the cardiovascular system produced in the Renaissance.

**Table:** 

Authors' roles & responsibilities	
ASR	Conception and study design; analysis and/or data interpretation; manuscript writing or critical review of its content; final approval of the manuscript
ETM	Conception and study design; analysis and/or data interpretation; manuscript writing or critical review of its content; final approval of the manuscript
